# ‘When They Drop in and They Are Crying’: Experience of University Academics Supporting Students with Mental Health Problems

**DOI:** 10.3390/healthcare13151792

**Published:** 2025-07-23

**Authors:** Oladapo Akinlotan, Christopher Wagstaff

**Affiliations:** 1 School of Nursing, Faculty of Health, Medicine & Social Care, Anglia Ruskin University, Bishop Hall Lane, Chelmsford CM1 1SQ, UK; 2 School of Nursing and Midwifery, University of Birmingham, Edgbaston, Birmingham B15 2TT, UK; c.wagstaff@bham.ac.uk

**Keywords:** university academics, university lecturers, university students, mental health problems, mental illness, higher education

## Abstract

**Background**: University academics are often the first points of contact for students experiencing mental health problems (MHPs) because of the pre-existing relationship between academics and students. **Aim**: The aim of this review is to explore the experience of university academics who have supported students with MHPs. **Methods**: This systematic review follows the Preferred Reporting Items for Systematic Reviews and Meta-Analysis guidance. Searches were conducted using six databases and were limited to peer-reviewed studies published in the English language between 2013 and 2023. **Results**: Thematic analyses identified three major themes: academics’ perceptions of mental health problems among students, the role of academics in supporting students and the academics’ perceived barriers to supporting students. **Conclusions**: Universities need to acknowledge the vulnerability of students’ mental health and prepare to respond appropriately. Improving the mental health literacy of university academics, and providing clarity about roles and mechanisms to support the mental health of university students will be crucial to achieve this.

## 1. Introduction

Mental health problems (MHPs) among university students have become a major issue [1,2]. University education represents huge opportunities for students but these also come with challenges, such as leaving home and family for the first time, increased independence and responsibility, academic stress, combining studies with personal and family life, financial worries and exposure to illicit substances and alcohol amongst many others [3]. All these issues represent significant changes to students and may create or exacerbate existing MHPs [1]. International students are those who are not nationals or citizens of the country where they study and they may refer to foreign students studying in higher education institutions outside of their own countries [4]. The term international students may be useful for immigration and fee-paying purposes [4]. On the other hand, home or local students refer to those who are citizens of the country where they study [4]. There are differences in the needs of international students when compared to home students [5]. International students’ needs include and are related but not limited to language barriers, education and cultural adjustments, accommodation and housing issues, financial problems, loneliness and isolation and immigration issues [5]. Although home students have unique challenges, they do not experience most of the problems faced by international students [5].

According to the World Health Organisation, about 35% of first-year students had at least one lifetime mental health disorder, while about 31% have at least one 12-month mental health disorder [6]. Confirming this worrying trend, professional and counselling services across universities have been reporting increasing demands for service and help from students experiencing various MHPs [7]. In addition to this, the recent global COVID-19 pandemic and its associated disruptions did not help the situation as the student population struggling with MHPs increased significantly [8]. There is a crisis in the mental of young people globally, exacerbated by COVID-19; the deterioration in the mental health of university students is an extension of this wider picture [8].

University academics are often the first points of contact for students experiencing MHPs because academics spend a lot of contact time with students and students feel that their lecturers or tutors can help them [9,10,11]. Although some academics with professional expertise (such as psychology, nursing, etc.) are able to use individual skills and professional experience to help support students who are struggling with MHPs [12], this support is limited in scope [9,11]. This limited support is mainly due to role confusion as academics and counsellors, and the lack of time to spend with struggling students [13]. Even though many university academics do not have professional or personal expertise to adequately support students that are struggling with MHPs, they feel under pressure to support their students with their MHPs [10,14].

It is important to stress that there are differences between professional counsellors and unprofessional academics who need to help students with mental health problems [15]. Professional counsellors are well-trained to support students who are experiencing mental health challenges and are usually available at counselling services across universities [16]. However, a majority of academics are not trained as counsellors or do not have any training in mental health and may find it difficult to support students who are struggling with their mental health [12]. Despite being unprofessional in counselling and mental health issues, students still prefer to speak to their lecturers about their mental health struggles [9].

Understanding the experiences of university academics who have supported students with MHPs is crucial to design and develop a support framework for university academics on how to effectively support/signpost students. The aim of this review is to explore the experience of university academics who have supported students with MHPs. This review uses a PEO (Population, Exposure and Outcome) framework, where population refers to university academics, exposure to students with MHPs and outcome to university academics’ experiences.

## 2. Methods

This is a systematic review of peer-reviewed qualitative and mixed studies that examined the experiences of university academics who have supported students with MHPs. The review follows the Preferred Reporting Items for Systematic Reviews and Meta-Analysis (PRISMA) guidance [17] and was published on the Prospero register (CRD42023445910).

### 2.1. Search Strategy

**Figure 1:**
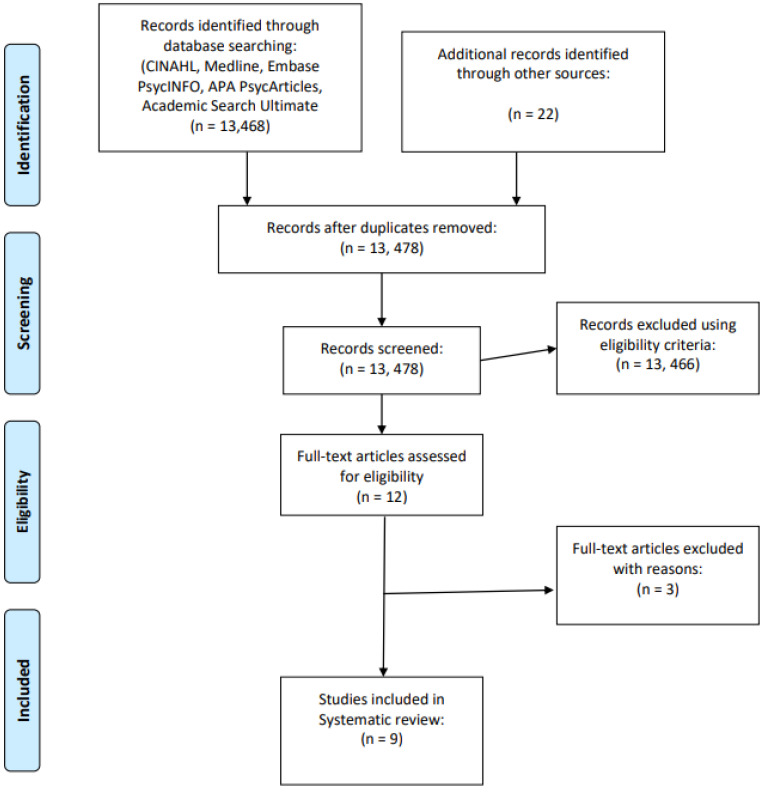
PRISMA flow diagram for this study.

The search strategy used combinations of medical subject heading (MeSH) terms and keywords, such as ‘lecturers’ or ‘academics’, ‘educators’, ‘mental health’ or ‘mental illness’ or ‘mental disorder’, ‘university students’ or ‘college students’ or ‘higher education students’. Searches were conducted using the following electronic databases: CINAHL Complete, MEDLINE Complete, PsycINFO, APA PsycArticles, Academic Search Ultimate and Embase. The searches were conducted between 16 and 18 December 2023. Additional searches of relevant journals and reference lists of included studies were conducted while relevant papers retrieved during the search were subjected to forward and backward searches by manually scanning their reference lists and the papers that cited them. The searches were limited to peer-reviewed journal articles published in the English language between 2013 and 2023 with available full text. Initial searches identified 13,468 studies and were screened to 12 studies for a full-text review (Figure 1). Eventually, a total of eight studies met the inclusion criteria (Table 1) and were included in the review. The two authors were involved in the selection and reviewing of studies. The first reviewer independently conducted search and selection while the second author reviewed 20% of the initial results (13,468) and the 12 studies identified for full-text review. Differences were resolved after discussion between the two reviewers. This is to mitigate selection biases.

### 2.2. Data Collection

Study characteristics of all the included studies were extracted into Microsoft Word (Table 2). The quality of the eight included studies was assessed using the Critical Appraisal Skills Programme [18] tool for qualitative research (Table 3). One reviewer conducted the first quality appraisal for all studies while the second reviewer independently conducted the second quality appraisal for four studies, which comprises 44% of all the studies considered. All disagreements were then resolved after further quality appraisal and discussion between the two reviewers. The quality assessment of the studies included was generally good; however, some studies did not adequately consider the relationship between the researcher and participants because data were collected anonymously (Table 3).

### 2.3. Data Analysis and Synthesis

All text from the ‘Results/Findings’ sections of each study were extracted and imported into Microsoft Word for data synthesis. Data synthesis was carried out using thematic synthesis [27]. Thematic synthesis was used as a systematic and transparent method to identify patterns and compare the main findings of all the included studies to add a new layer of interpretation. All the studies were carefully read to ensure familiarisation with the contents and to develop initial codes by using a comparative approach. Initial coding and descriptive coding were carried out by the first reviewer, and these were independently checked by the second reviewer. Initial codes were collapsed into descriptive codes, and these were then collapsed into initial themes and subthemes. These were carried out by the first reviewer and were independently checked by the second reviewer. These initial themes and subthemes were compared and analysed to identify the final major themes and subthemes presented in the results. The first reviewer identified the final major themes and subthemes, and these were independently checked by the second reviewer. The codes, themes and subthemes were agreed upon between the two reviewers and differences at any stages were resolved after discussion between the two reviewers.

## 3. Results

**Figure 2:**
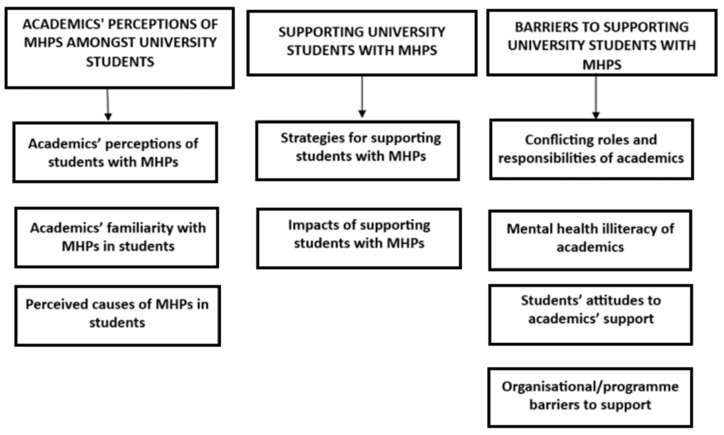
Three major themes and nine subthemes identified from the review.

Three major themes and nine subthemes were identified (Figure 2): 1. Academics’ perceptions of MHPs amongst university students (academics’ perceptions of students with MHPs, academics’ familiarity with MHPs in students and perceived causes of MHPs in students). 2. Supporting university students with MHPs (strategies for supporting students with MHPs and impacts of supporting students with MHPs). 3. Barriers to supporting university students with MHPs (conflicting roles and responsibilities of academics, mental health illiteracy of academics, students’ attitudes to academics’ support and organisational/programme barriers to support).

### 3.1. Academics’ Perceptions of MHPs Amongst University Students

This theme discusses the perceptions of university academics regarding MHPs amongst university students using three subthemes: prevalence of students with MHPs, academics’ familiarity with MHPs in students and perceived causes of MHPs in students.

#### 3.1.1. Academics’ Perceptions of Students with MHPs

University academics have witnessed a significant increase in the number of students with MHPs [19]. The widespread prevalence of MHPs amongst students means that most university academics have encountered students with MHPs [26]. Academics with more teaching experience are likely to have taught students with MHPs [21,22,23].

*‘I have witnessed a spurt in the number of students who have approached me regarding wanting to share personal difficulties… asking me for referrals to outside experts’*

[19].

Students approached academic staff to share their MHPs ranging from feelings of anxiety and emptiness to trying to commit suicide [19,21].

*‘He wrote a long email to me talking about the darkness in his life, shared about having tried to commit suicide earlier and the fact that he had completely stopped his medication in the last one month’*

[19].

Farrer and Gulliver [21] observed that students are more likely to confide in academic staff in health and medical sciences and also in course convenors/leaders. In general, students were initially reluctant to share their MHPs but they gradually became more open when they began to trust the academic staff [19].

*‘Gradually,… he started opening up and shared about facing mental health difficulties since his school days’*

[19].

Many students seek support for their MHPs from their lecturers/tutors [24], partly because they believe that only the academic staff can understand and support them [19].

*‘He told me that only I could understand him fully and he really appreciated my support’*

[19].

Some students kept in touch with academic staff and continued to meet them for support and counselling [19]. Others went away for treatment and returned to complete their courses but some quit their courses because of MHPs [19].

*‘I conveyed my empathy and respected his decision to leave the course and wished him good luck’*

[19].

#### 3.1.2. Academics’ Familiarity with MHPs in Students

In general, university academics are very familiar with depression, anxiety disorder, and attention deficit disorder, but not familiar with personality disorders, schizophrenia, paranoia [20].

*‘Students with depression and anxiety is the most common… quite a few of them have already been diagnosed’ (P9—post-1992, M&HS)*

[26].

The common signs, symptoms and related MHPs issues that university academics observed amongst students include excessive absences, lack of concentration, late submission of assignments, major or unusual change in appearance and sudden drop in academic performance [19,20,25,26].

*‘The season and clothes were not matching, wearing a mask all the time (FG3)’*

[25].

#### 3.1.3. Perceived Causes of MHPs in Students

University academics believe that demands of academic programmes, course contents and academic criticism can have negative impacts on the mental health of students [24].

*‘Students can gradually lose confidence in themselves, and they may have to repeat the year … they started to live alone, they can no longer eat properly (FG2)’*

[25].

*GA: ‘I teach palliative care, so I tend to get… people come up to me at the end of lectures and talk to me about bereavements and such and so it is an issue that I do, sort of, it is something that happens quite a lot’*

[24].

Some health-related students who have to complete placement (clinical practice) as part of their course requirements can also be vulnerable. Placement can be potentially problematic for students because it can put students’ mental well-being under further pressure [24].

*‘… many students get messed up when they went to clinical practice and confronted people (FG3)’*

[24].

*GA: Sometimes [mental health difficulties get] identified in practice and you’ll get a phone call or frantic email with concerns about a student in practice. I think that is one of my most difficult experience’*

[24].

University academics also noticed genetic/hereditary disposition to MHPs [19,25].

*‘… suicide issue and depression are really big [in families] as MHPs. (FG3)’. Relationships between a child and parent/s … I wonder if they drag mental [health] problems from childhood (FG1)’*

[25].

Isolation in society, bullying, harassment and the resulting withdrawal, lack of face-to-face contact are other observed issues [25].

*‘It is a stressed society’ (FG2)…’a nuclear society with nuclear families [and] it is the loneliness (FG1)’*

[25].

*‘…Discussion on depression and mental problems from bullying or harassment are important (FG2)’*

[25].

Academic staff believe that there is a need to differentiate between MHPs and normal emotions and the university should be helping students to recognise “normal” psychological demands and deal with them appropriately [26].

*‘There is a difference between stress and MHPs that needs to be relayed to both staff and students (SR—Russell Group, ET&NS)’. ‘Everyone gets anxious … this is different to a clinical diagnosis of anxiety (P2—other pre1992, ET&NS)’*

[26].

### 3.2. Supporting University Students with MHPs

This theme discusses how university academics support students with MHPs using two subthemes: strategies for supporting students with MHPs and impacts of supporting students with MHPs.

#### 3.2.1. Strategies for Supporting Students with MHPs

Overall, most academics are willing to provide support to students with MHPs [20,21,26].

*‘I am happy to talk with students, be with them during difficult times, and sort out practical problems (SR—Russell Group, ET&NS)’*

[26].

Staff from health and medical sciences are more likely to initiate conversation with students with MHPs [21]. However some academic staff feel less confident about talking to, listening to and reassuring students with MHPs and most staff feel unconfident when talking to students about suicidal thoughts [22].

Overall, university academics provide three levels of support to students with MHPs: emotional (personal) support, instrumental (programme) support and referral (professional) support [19,22].

Academic staff provided emotional support by showing empathy to students, providing a listening ear and conducive environment, having supportive conversation, providing counselling, pastoral care and encouraging student to contact them if needed [19,22].

*‘Said I was sorry she was having difficulties this semester…if I could do anything to help her successfully complete the semester to let me know’*

[22].

Academic staff provided encouragement and recommendations regarding self-help, coping strategies, importance of adhering to treatment plans and involved students’ friends and family [19,22].

*‘I suggested options for the student to consider in terms of accessing appropriate help and offered to facilitate that if necessary’*

[22].

*‘I contacted his family and requested them to come to take him to the native place’*

[19].

Providing emotional support was higher in staff who initiated conversation with students compared to those who just taught them [22].

Secondly, academic staff provided instrumental support by adjusting teaching, assessment and deadlines, providing personal time and assistance and accommodating formal requests (e.g., recording lectures, private test/exam room, exception from exam) to accommodate students with MHPs [19,20,22].

*‘Told them that I was amenable to adjusting deadlines for assessment or requirements of the course’*

[22].

Staff involved others within the university in managerial positions, or those with a duty of care to the students to notify them, for seeking advice or information about where to direct students [19,22,26].

*‘I discussed it with the director of teaching and sought advice and assistance’*

[22].

Referral/professional support: University academics made referrals within and outside the university [19,20,22].

*‘I refer them to the disabilities service centre (DSC), who can make an assessment and identify where else they should be referred’*

[22].

Staff contacted other professionals or services on a personal basis on behalf of students [19,20].

*‘Asked for their permission to make them an emergency appointment with the Counselling Centre and made that appointment in their presence. Offered to escort them to that appointment’*

[22].

Some academics believed that online mental health resources are useful while female staff are more likely to believe that online resources are as useful as face-to-face [21]. Some staff are willing to recommend online resources to students if they are endorsed by the university and if students use them as a first step toward seeking professional help [21].

*‘I would be willing to provide online resources for students as a reference that will assist them in engaging professional support. Online self-help programmes are valuable if the person has already sought professional assistance and only requires additional assistance to help guide them’*

[21].

On the other hand, some academics do not believe that online mental health resources are a credible treatment option and offer the same quality as face-to-face services [21]. Hence, they are less likely to recommend online mental health resources to students [21]. Some academics believe that recommending online interventions would de-value students, imply that students are not worthy of face-to-face care, encourage isolation and discourage students from seeking professional help [21].

*‘If I was to suggest going online to seek help, I would think this would in part reinforce the idea that they [students] are not valued. I would be more concerned that students would continue to feel isolated whilst seeking online help’*

[21].

Some academics felt that students might react negatively to them or target them if they recommend online resources to students while others expressed concerns about the quality of online interventions, perceived dangerousness, its generic nature, and inability to deliver effective help [21].

*‘I would worry it might lead to problematic self-diagnoses and may expose students to harmful online communities. I do not believe that online courses can substitute for face-to-face services’*

[21].

Brockelman and Scheyett [20] discussed the use of PAD (psychiatric advance directive) by students to help academics support them during mental health crises. PAD helps staff to know how to support a student during crisis so that they can receive consistent care and can lower the risk of injury or harm to a student during a mental health crisis [20].

*‘Students should be more involved and proactive in managing their mental health.’ ‘They should be empowered to assert their own wishes when they are well.’*

[20].

On the other hand, academic staff had concerns about the good judgment needed to make an appropriate PAD, conflict between a student’s wishes and professional judgement, confidentiality, logistical problems [20].

*‘It has been my experience that when a person with a mental illness is doing well could be when they are in most danger of deciding to discontinue their medications, so I am a bit conflicted about a student writing what meds they want to be given (should their condition deteriorate) while they are feeling good because that is when many decide they do not really need meds any longer’*

[20].

#### 3.2.2. Impacts of Supporting Students with MHPs

Supporting students with MHPs affected some academics’ mental health and some felt distressed during or after contact with students with MHPs [26].

*‘If you’re dealing with students who are experiencing paranoia or depression, then managing that student in the classroom is going to have an impact on your own mental health and sense of well-being and confidence (P1—post-1992, SS)’*

[26].

Academic staff witnessed increased personal concerns about students with MHPs and worried that students might harm themselves [19].

*‘It is terrifying feeling responsible for a suicidal student and worrying if you have given the right advice … (SR—other pre-1992, M&HS)’*

[26].

Supporting students with MHPs also impacted on academics’ wider roles [26]. Many academics experienced difficulties (disruption to other students, inappropriate communication, unreasonable complaints, threatening behaviour) while working with students with MHPs [26].

*‘ …it is also incredibly time-consuming and disruptive to work (SR—other pre-1992, M&HS)’*

[26].

### 3.3. Barriers to Supporting University Students with MHPs

This theme discusses the barriers that university academics face when supporting students with MHPs using four subthemes: conflicting roles and responsibilities of academics, mental health illiteracy of academics, students’ attitudes to academics’ support and organisational/programme barriers to support.

### 3.4. Conflicting Roles and Responsibilities of Academics

For healthcare academics, there are two professional roles and identities (academic and clinician) [24], which they use to support students with MHPs [19]. Sticking to the boundaries of the role of academic can be personally challenging for many academics [24].

*GA: ‘But when they drop in and they are crying…with no appointment, so you go back to that, what feels very natural to me, the nurse–client relationship, not the teacher–student relationship’*

[24].

*P5: ‘When I stopped being a practitioner to become an academic, I was worried that I’d stop being a practitioner and end up becoming [just] an academic and that hasn’t happened at all. It’s just like I am doing practice really’*

[24].

Some academic staff are unclear about their duties/roles as regards assisting students with MHPs [22] and there is lack of clarity on what an academic’s role should be [26]. Many academic staff do not believe or feel that it is part of their teaching role to manage or assist students with MHPs [21,22].

*GA.3: ‘One of them (colleagues) was very clear to say to her colleagues, ‘you are their personal tutor, you are not their therapist’ I think we struggle’*

[24].

*‘I do not want to deal with any student health issues. I don’t want a student to act on my advice, simply because I am in a position of authority, and then later blame me if that advice was inappropriate’*

[21].

Some academics feel that it could be an awkward or uncomfortable conversation talking with students about their MHPs [21].

*‘I would be concerned about making them uncomfortable by indicating or suggesting that I think they would need help. It might be a bit awkward. I wouldn’t want to embarrass the student’*

[21].

Academic staff observed blurred roles and boundaries and problems maintaining clarity about the boundaries of roles [24].

*P14: ‘I’m not here as a practising occupational therapist, you know… but I have that knowledge and that background that helps me pick up on those things’*

[24].

Many academic staff feel that they do not feel qualified to recommend online resources to students [21].

*‘… there are many things that academics don’t understand even if we are involved … it’s best for the specialist to support them… (FG2)’*

[25].

*‘I am not a medical doctor or a social worker or a carer, it is not my job to support people with mental health issues (SR—Russell Group, A&H)’*

[26].

Very few academics did nothing when they feel it is not their role to help [22].

*‘Nothing. The issue was (and should be) dealt with by more qualified people who should advise the lecturer if they need to do anything’*

[22].

*‘I was not sure what to do’*

[22].

On the other hand, academics did something when approached by students with suicidal thoughts [22].

*P5: ‘When I stopped being a practitioner to become an academic, I was worried that I’d stop being a practitioner and end up becoming [just] an academic and that hasn’t happened at all. It’s just like I am doing practice really’*

[24].

Relationship between academics and students can create a challenge for boundaries [24]. For example, strong positive relationships between academics and students can make some students reluctant to accept signposting or support from other services [24].

*P9: ‘The thing I find most difficult is that we do have a good relationship with our students… when they come to you, they don’t really want you to send them somewhere else’*

[24].

### 3.5. Mental Health Literacy of Academics

The majority of academic staff do not feel sufficiently informed to respond appropriately to students with MHPs [21,22]. Academics’ lack of knowledge and skills in supporting students with MHPs is a sector-wide issue and consistent across types of institution and subject area.

*‘I don’t know anything about them and do not feel qualified to be able to direct students to reputable ones’*

[21].

A minority of academic staff feel that their institution had adequately prepared them for working with students with MHPs and these academics are sufficiently informed to respond appropriately to students [21]. On the other hand, a significant number of academics had not received training on how to respond to students with MHPs [22,26]. The overall level of training received by academic staff varies but is still inadequate [26]. Some academic staff are concerned about their lack of knowledge to support students with MHPs [25]. Not all academic staff were aware of online resources for MHPs, but the majority are willing to learn more about online mental health resources [21].

*‘I’m not trained, I’m not a professional’*

[26].

Academics from a health/behavioural science background had significantly higher levels of literacy about depression [23]. Academic staff with higher literacy about depression are more likely to have initiated a conversation with a student, felt sufficiently informed to respond appropriately to students and demonstrated significantly lower levels of stigmatising attitude to depression [23].

*GA.1: ‘It is difficult because we are not just academic staff, we are on a professional register as well and it is difficult to keep these two things apart…’*

[24].

The majority of academic staff were unsure about university written policy on how to respond appropriately to students with MHPs, while some academic staff believed there was no formal training available [22]. The majority of academic staff found it satisfactory to receive training (face-to-face, online, printed manuals) to assist students with MHPs [22,26]. Academics desired training in depression, generalised anxiety, exam anxiety, self-harming, autism, eating disorders, substance use and for making referrals [22]. Academic staff requested that the delivery of the training be clear and brief and discussed issues around the appropriateness of presenters [22].

*‘I have generally found such sessions unhelpful. The advice offered tends to be trite and/or bureaucratic. Presenters seldom have knowledge of the reality of the classroom’*

[22].

### 3.6. Students’ Attitudes to Academics’ Support

Students need to recognise when they need professional help and take responsibility for assessing available support [25,26]. Unfortunately, some students do not know that they have MHPs [20].

*‘They may not even realise whether they are strange or normal…a ‘[lack of] self-awareness (FG2)’*

[25].

*‘Even when they (students) have poor mental health, they don’t take the initiative to go to the hospital outside unless it is so bad (FG1)’*

[25].

Barriers to students seeking support observed by academic staff include denial, fear of stigma and being judged, fear of being labelled, concerns about confidentiality and completing the course, students’ prejudices, and students having problems connecting with university staff/academics [20,25].

*‘When the student comes to the counsellor’s office, … I think that they worry about ‘the eyes’ around them … students don’t want to be seen with ‘such eyes’ (FG2)’*

[25].

*‘So, it may be pride … or they may be embarrassed … and being ashamed as well … and when the people label the student, in the form of ‘he/she is such a person (FG2)’*

[25].

### 3.7. Organisational Barriers to Support

Academic staff do not have sufficient time to support students with MHPs [26].

*‘… even though I want to take time and thoroughly listen to this student … we cannot have time and a place in the environment… (FG2)’*

[25].

Academic staff experienced problems with supporting students on placement as responding to students’ MHPs during placement can be more difficult [24].

*‘… many students get messed up when they went to clinical practice and confronted people. (FG3)’*

[24].

*GA: ‘Sometimes [mental health difficulties get] identified in practice and you’ll get a phone call or frantic email with concerns about a student in practice. I think that is one of my most difficult experiences’*

[24].

## 4. Discussion

The aim of this review is to explore the experience of university academics who have supported students with MHPs. Using three major themes and nine subthemes, this review has noted the wide spread of MHPs amongst student populations, the roles of academics in supporting students and the barriers they encounter while doing this. The studies included in this review highlighted the widespread prevalence of MHPs amongst university student populations, which increases the likelihood of academics coming in contact with these students. It has been reported that there has been an increase in the report of diagnosed and undiagnosed cases of MHPs in the general population and this increase had led to a corresponding increase in MHPs amongst the student population [28]. Hence, it is not surprising that universities are seeing increases in cases of MHPs amongst students [1]. Strangely, increases in MHPs amongst university students has not led to a corresponding increase in mental health literacy of university academics [29]. It is either that the universities are not prepared for this increase or they lack the resources to respond appropriately [29]. While the discrepancies between the increased mental health needs and coverage at universities are alarming, this is not surprising [8]. This reflects the current situation regarding the mental health crisis: the substantial increase in MHP prevalence with the long-term gap between mental health needs and resources dedicated to mental health prevention, attention, and care [7].

Studies from this review have shown that, despite being a great source of opportunities for students, the university can also be a place where the mental health of students is subjected to huge amount of pressure [28]. Some explanations for this have been suggested: leaving home, moving abroad, becoming adults, leaving independently, academic and financial demands, etc., are some of the reasons why university attendance makes many students vulnerable to MHPs [1]. Universities need to acknowledge these vulnerabilities and prepare to respond appropriately and in a timely manner to provide support for MHPs amongst students [30]. Making university education, culture and systems flexible for students to navigate will go a long way to minimise unnecessary stress on students’ mental health [12].

What the studies from this review have shown is that, overall, university academics are open to supporting students with MHPs but they are more or less incapable of successfully doing this for many reasons [9]. One of the ways to equip academics to be able to support students is to provide clarity regarding their role in providing support to students [12]. Studies from this review noted the confusion and clash of roles of academics as a major barrier to supporting students with MHPs. The tag of ‘personal tutor’ or ‘personal and development tutor’, etc., is commonly used in many universities and comes with various responsibilities, including pastoral support [31]. Despite this tag, many university academics are unclear about the limit of their involvement when dealing with students with MHPs [12].

Studies from this review have identified mental health literacy of university academics as another major issue preventing them from providing adequate support to students [29]. University academics need to receive appropriate and relevant training to help them support students adequately and the university should facilitate this [29]. Studies from this review have shown that students’ attitudes to support are among the barriers to supporting university students with MHPs. As a result, the mental health literacy campaign needs to be extended to students as well, as this will help them to identify MHPs, know when to seek help and cooperate with university academics as they support them [32]. Studies from this review noted the negative impacts on the mental health of university academics as they support students with MHPs, as well as the requirements of universities to provide support to academics to mitigate any negative impacts of supporting students with their own mental health [12]. In conclusion, many factors need to be considered when considering academics who support students with MHPs. These include but are not limited to the ranking of the universities, the subject or major of the academics (e.g., psychology lecturers or computer science lecturers), the position of the academics (e.g., lecturers or professors), and the mother tongue of the academics and the students [1,8].

### 4.1. Strengths and Limitations

To the best of the authors’ knowledge, this study represents the first qualitative systematic review and synthesis of the most current evidence on the experience of university academics supporting students with MHPs globally. The review has conducted an extensive literature search covering the most relevant healthcare databases. As the review involved studies from several countries, the findings can be useful and applicable in other countries around the world. Included studies were limited to peer-reviewed studies to ensure validity of the findings, while included studies were subjected to quality appraisal process using Critical Appraisal Skills Programme (CASP [18]).

This review has some limitations. Studies published prior to 2013 may be useful but have been excluded to focus on the most current trends. Studies published in other languages apart from English may also be useful, but these have been excluded because interpretation resources were not available for this review.

### 4.2. Recommendations

Based on the findings of this review, the following recommendations are suggested:

- Universities need to provide clarity about ‘academic’ and ‘clinical’ roles to university academics to help them provide timely and appropriate support to students with MHPs.
- Universities need to embark on efforts to improve mental health literacy of university academics and university students.
- Universities need to provide a workable mechanism to support the mental health of university academics to mitigate any negative impacts of supporting students with MHPs.

## 5. Conclusions

MHPs among university students have become a global concern. University academics are usually the first points of contact for students experiencing MHPs, because academics spend a lot of contact time with students and students feel that their lecturers can help them with their MHPs. Eight studies met inclusion criteria and were analysed using thematic synthesis. Three major themes and nine subthemes were identified: 1. Academics’ perceptions of MHPs amongst university students (academics’ perceptions of students with MHPs, academics’ familiarity with MHPs in students and perceived causes of MHPs in students). 2. Supporting university students with MHPs (strategies for supporting students with MHPs and impacts of supporting students with MHPs). 3. Barriers to supporting university students with MHPs (conflicting roles and responsibilities of academics, mental health illiteracy of academics, students’ attitudes to academics’ support and organisational/programme barriers to support). The widespread prevalence of MHPs amongst students means that most university academics have encountered and supported students with MHPs. Although university academics are open to supporting students with MHPs, many of them are incapacitated by this. Conflicting roles and mental health illiteracy of academics and students’ attitudes are the major barriers. Universities need to acknowledge the vulnerability of students’ mental health and prepare to respond appropriately. Improving mental health literacy and providing clarity about roles and mechanisms to support the mental health of university academics will be crucial to achieve this.

## Figures and Tables

**Table 1:** The eligibility criteria for the studies considered.

	Inclusion	Exclusion
Study design and characteristics	Primary research studies (that include primary data) using any qualitative design (pure or mixed methods); Peer-reviewed journal articles only; Studies with available full text only; English-language only; Any year from 2013 to 2023.	Quantitative studies only Articles reporting no primary data, e.g., protocols, editorial, commentaries, conference abstracts; Secondary research, e.g., reviews; Non-English-language studies; Studies published prior to 2013.
Population/ participants	Studies that considered university academics (lecturers, professors or educators) at any university across the world; Participants of any age, gender, race or sexuality.	Studies that considered university non-academics; Studies that considered university academics together with university non-academics in a university.
Exposure	Studies that examined students’ mental problems; Studies that considered students with diagnosed, undiagnosed or suspected mental disorders or illnesses; Studies that considered students with either pre-existing condition or new diagnosis at university.	Studies with students that do not have mental illness or disorder (diagnosed, undiagnosed or suspected); Studies with students that talked generally about mental well-being.
Outcome	Qualitative studies that examined university academics’ experiences and views of supporting students with diagnosed, undiagnosed or suspected mental disorders or illnesses; Qualitative studies that acquired quantitative data as part of the study; Qualitative studies that included and separately reported on the experiences of university academics who supported students with diagnosed, undiagnosed or suspected mental disorders or illnesses.	Qualitative studies that did not examine university academics’ experiences and views of supporting students with mental disorders or illnesses; Qualitative studies that did not separate the reported experiences of university academics who supported students with diagnosed, undiagnosed or suspected mental disorders or illnesses.

**Table 2:** Characteristics of the reviewed studies. N: number of participants. A: Age (mean), G: gender (Male/Female/Others), T: teaching role, P: programme, UG: undergraduate, PG: postgraduate, E: experience in years (mean), D: discipline, N/A: Not Applicable.

Author/ Country	Aims	Participants’ Age and Gender	Participants’ Professional Characteristics	Methods	Data Analysis
[19] India	To contextualise students’ challenges and gauge its implications for the social work programme	N: 1 A: N/A G: 0/1	T: lecturer, fieldwork supervisor P: PG E: N/A D: Social work	Case vignettes	Case narratives
[20] United States of America	To provide information on faculty knowledge of mental health problems in students, their use of available accommodations and strategies, and their willingness to accept psychiatric advance directives.	N: 168 A: 47 G: 82/86	T: lecturers P: UG and PG E:14 D: N/A	Open-ended questionnaire	Thematic analysis
[21] Australia	To ascertain teaching staff experiences of providing support to students with mental health problems and their awareness and willingness to recommend online mental health resources to students.	N: 224 A: 41.6 G: 113/110/1	T: lecturers, tutors, course convenors and research supervisors P: UG and PG E: 10 D: Sciences, Business, Law	Mixed-method including online questionnaire	Thematic and statistical analyses
[22] Australia	To investigate university teaching staff experiences of, and training needs around, assisting students with mental health problems.	N: 224 A: 41.6 G: 113/110/1	T: lecturers, tutors, course convenors and research supervisors P: UG and PG E: 10 D: Sciences, Business, Law	Mixed-method including online questionnaire	Thematic and statistical analyses
[23] Australia	To investigate university teaching staff literacy and the stigma they attach to depression and assess the influence of this on their assistance of students with mental health problems.	N: 224 A: 41.6 G: 113/110/1	T: lecturers, tutors, course convenors and research supervisors P: UG and PG E: 10 D: Sciences, Business, Law	Mixed-method including online questionnaire	Thematic and statistical analyses
[24] United Kingdom	To explore how academics on nursing and healthcare programmes are managing their roles and responsibility in relation to student mental health.	N: 14 A: N/A G: 2/12	T: lecturers, tutors, programme leads, P: N/A E: N/A D: Nursing, medicine and healthcare	Semi-structured individual interviews and focus groups	Thematic analysis
[25] Japan	To explore the attitudes, beliefs, knowledge and practices of university academics towards health science students with psychological/mental health issues.	N: 15 A: 44.2 G: 1/14	T: lecturers, professors P: UG and PG E: 6.8 D: health science	Semi-structured interview with focus groups	Thematic analysis
[10] Australia	To explore the nature, extent, and impacts of interactions between university staff engaging with students who disclose that they are experiencing a mental health challenge.	N: 22 A: N/A G: N/A	T: academic staff P: N/A E: N/A D: N/A	Semi-structured interview	Thematic analysis
[26] United Kingdom	To explore how academics in different types of institution and subject areas perceive mental health problems amongst students, and how have they experienced working with students with mental health problems.	N: 130 A: N/A G: N/A	T: lecturers and research supervisors P: N/A E: N/A D: N/A	Online survey and semi-structured interviews	Statistical and thematic analyses

**Table 3:** Quality assessment of the included studies using CASP [18]. Y: Yes, N: No, V: Valuable.

	Anand [19]	Brockelman and Scheyett [20]	Farrer, Gulliver [21]	Gulliver, Farrer [22]	Gulliver, Farrer [23]	Hughes and Byrom [24]	McAllister, Wynaden [10]	McMaster [25]	Spear, Morey [26]
Was there a clear statement of the aims of the research?	Y	Y	Y	Y	Y	Y	Y	Y	Y
Is a qualitative methodology appropriate?	Y	Y	Y	Y	Y	Y	Y	Y	Y
Was the research design appropriate to address the aims of the research?	Y	Y	Y	Y	Y	Y	Y	Y	Y
Was the recruitment strategy appropriate to the aims of the research?	Y	Y	Y	Y	Y	Y	Y	Y	Y
Was the data collected in a way that addressed the research issue?	Y	Y	Y	Y	Y	Y	Y	Y	Y
Has the relationship between researcher and participants been adequately considered?	Y	N	N	N	N	Y	N	N	N
Have ethical issues been taken into consideration?	Y	N	Y	Y	Y	Y	Y	Y	Y
Was the data analysis sufficiently rigorous?	Y	Y	Y	Y	Y	Y	Y	Y	Y
Is there a clear statement of findings?	Y	Y	Y	Y	Y	Y	Y	Y	Y
How valuable is the research?	V	V	V	V	V	V	V	V	V

## Data Availability

The original data presented in this study are openly available in [healthcare databases].
